# Diosmin Attenuates Age-Associated Vascular Oxidative Stress and Endothelial Dysfunction in Aged Rats

**DOI:** 10.3390/foods15162779

**Published:** 2026-08-07

**Authors:** Giulia Valdiserra, Clelia Di Salvo, Matteo Fornai, Carolina Pellegrini, Letizia Campigli, Alessandro Mengozzi, Federica Cappelli, Emiliano Duranti, Chiara Ippolito, Rocchina Colucci, Nunzia Bernardini, Agostino Virdis, Luca Antonioli

**Affiliations:** 1 Department of Clinical and Experimental Medicine, University of Pisa, 56126 Pisa, Italy; fcappelli1996@gmail.com (F.C.); agostino.virdis@unipi.it (A.V.); luca.antonioli@unipi.it (L.A.); 2 Department of Pharmaceutical and Pharmacological Sciences, University of Padova, 35131 Padova, Italy

**Keywords:** diosmin, inflammaging, vascular aging, oxidative stress

## Abstract

Vascular aging is driven by a self-sustaining interplay between oxidative stress and chronic low-grade inflammation, collectively referred to as inflammaging. Diosmin, a citrus-derived flavonoid with antioxidant and anti-inflammatory properties, has not previously been investigated in this context. Young (10-week-old) and aged (40-week-old) male Sprague–Dawley rats received diosmin (50 mg/kg/day) or vehicle for 3 months. Vascular function was assessed in mesenteric resistance arteries using pressure myography, while oxidative stress was evaluated by dihydroethidium and mitoSOX staining. Circulating malondialdehyde (MDA), tumor necrosis factor (TNF), lipopolysaccharide-binding protein (LBP) vascular expression of *Nos3*, *Gpx1*, *Sod2* and *Xdh* were measured. Aging was associated with impaired endothelium-dependent vasorelaxation, increased vascular DHE and MitoSOX fluorescence and elevated circulating MDA and LBP levels. Chronic diosmin supplementation partially restored endothelial function. The improvement in endothelial function was consistent with a partial preservation of NO-dependent endothelial function, although NO production and bioavailability were not measured directly. Diosmin also significantly reduced vascular DHE and MitoSOX fluorescence in aged animals. These effects were accompanied by decreased plasma MDA and LBP levels, whereas TNF remained unchanged. Diosmin did not significantly affect *Nos3*, *Gpx1* or *Sod2* gene’s expression, but it attenuated the age-related upregulation of *Xdh*. Overall, diosmin attenuated age-associated vascular oxidative stress and partially improved endothelial function in aged rats. Although the reduction in circulating LBP suggests a possible effect on selected systemic signals associated with endotoxin exposure, additional inflammatory markers and mechanistic studies will be required to determine whether diosmin modulates vascular inflammaging.

## 1. Introduction

The term inflammaging—a fusion of “inflammation” and “aging”—refers to findings from various preclinical and clinical studies that highlight a close relationship between the processes that lead to aging and the presence of a persistent and systemic mild inflammatory response, without visible clinical symptoms (latent), but which can be the basis for several chronic degenerative diseases, commonly observed in the elderly [1]. The condition of inflammaging seems to result from a progressive reduction in the biological reserve mechanisms that allow the body cells to counteract changes and challenges to the tissue homeostasis. These changes depend on the environmental exposure and chronic accumulation of inflammatory damages over the course of an individual’s existence, inducing an impairment of the biological reserve higher than that associated solely with aging [1].

The condition of inflammaging is characterized by high circulating levels of pro-inflammatory markers, including interleukin (IL)-1β, IL-6, IL-8, IL-13, IL-18, C-reactive protein, interferon (IFN)-α and IFN-β, transforming growth factor (TGF)-β, tumor necrosis factor (TNF) and serum amyloid type A [1,2]. Although initially these inflammatory alterations were considered an inevitable consequence of aging, an increasing body of experimental and human data suggests that they may represent also the primary causes of aging, thus configuring the occurrence of a vicious circle of events where aging represents the source of inflammation and, at the same time, the pathological target of its consequences [3].

Current evidence indicates that chronic low-grade inflammation during aging is driven by multiple interconnected mechanisms, including cellular senescence and the senescence-associated secretory phenotype (SASP), mitochondrial dysfunction, oxidative stress, impaired autophagy, innate immune dysregulation, alterations in gut barrier integrity and microbiota composition and accumulation of endogenous danger-associated molecular patterns (DAMPs). Rather than acting independently, these mechanisms reinforce one another through self-sustaining feed-forward loops, making tissue-specific therapeutic targeting particularly relevant [1,2,4].

Although the mechanisms underlying the above vicious circle remain largely to be defined, the increase in oxidative stress with advancing age, resulting from an impairment of antioxidant defenses and an increase in the production of oxygen free radicals, seems to play a central role as mediator of the relationship between inflammation and cellular aging. Indeed, an increase in the production of oxygen free radicals, especially of mitochondrial origin, represents an important stimulus to the activation of the inflammatory response [5], and, simultaneously, it can damage the major cellular macro-elements (proteins, lipids, nucleic acids). In the early stages, this damage can be repaired by cellular defense mechanisms. However, its chronicization makes such intracellular defenses insufficient, leading to the malfunctioning of enzymes and cellular barrier structures as well as to altered gene expression that are believed to be at the basis of the evolution of organic aging [6]. At the same time, the inflammatory response, sustained by the increase in oxidative stress, is almost invariably associated with an increase in the production of oxygen free radicals, thus favoring a pro-oxidant environment that could support the onset and persistence of a chronic inflammatory process [1,2].

The tight relationship between oxidative stress, inflammation and aging is confirmed by the scientific literature that has clearly identified inflammation and oxidative stress as the main actors underlying the development of chronic degenerative diseases commonly occurring in the elderly, such as Alzheimer’s, type II diabetes, and cardiovascular diseases [1,2].

Recently, diosmin, a citrus-derived flavone glycoside widely used in the treatment of chronic venous disorders, has emerged as a promising agent for several conditions characterized by oxidative stress and chronic inflammation, including those associated with aging [7,8]. Diosmin is naturally present in citrus fruits and is generally produced at the industrial level from citrus-derived hesperidin through a dehydrogenation process, followed by purification and standardization. Since the manufacturing procedures and formulation may influence the physicochemical characteristics and oral bioavailability of the final product, the characterization of the diosmin-containing ingredient is relevant when interpreting preclinical and clinical findings. Diosmin exerts anti-inflammatory effects by inhibiting NF-κB signaling and reducing the expression of several pro-inflammatory cytokines, while its antioxidant properties include free-radical scavenging and attenuation of oxidative stress biomarkers [7,8]. However, despite these well-established biological activities, no studies have specifically investigated the potential of diosmin to counteract vascular inflammaging. Diosmin was selected because, aside from its well-established clinical use, it combines antioxidant, anti-inflammatory and vascular-protective properties together with favorable pharmacokinetic characteristics [3,5], making it an attractive candidate for investigating the pharmacological modulation of vascular aging.

## 2. Materials and Methods

### 2.1. Experimental Design and Administration

Aged (40 weeks old, 500–600 g of body weight) and young (10 weeks old, 200–225 g of body weight) Albino male Sprague–Dawley rats were purchased from ENVIGO S.r.l (San Pietro al Natisone, Italy) and employed throughout the study. Animals had free access to standard laboratory chow and tap water and were given at least 1 week to acclimatize before the beginning of experimental procedures.

They were housed, three in a cage, in temperature-controlled rooms at 22–24 °C and 50–60% humidity with a 12-h light cycle. Animal care and handling were in accordance with the provisions of the European Community Council Directive 210/63/UE, implemented by the Italian Government. The experiments were approved by the Ethical Committee for Animal Experimentation of the University of Pisa and by the Italian Ministry of Health (authorization n 1023/2023-PR). Animals were monitored daily to assess any sign of distress and all efforts were made to minimize animal suffering.

Aged rats were considered a natural model of vascular inflammaging. Thirty-two animals were randomly assigned to four experimental groups: (a) young + vehicle (*n* = 8); (b) young + diosmin (50 mg/kg/day; *n* = 8); (c) aged + vehicle (*n* = 8); and (d) aged + diosmin (50 mg/kg/day; *n* = 8). The dose of 50 mg/kg/day was selected on the basis of previous in vivo evidence demonstrating the cardiovascular and metabolic effects of diosmin at this dose in rats [9]. The present study was designed to assess the long-term vascular effects of a previously established biologically active dose rather than to define a dose–response relationship. Young vehicle-treated animals represented the physiological reference group, whereas aged vehicle-treated animals served as the untreated aged control group for evaluating the effects of diosmin treatment. Animals received diosmin or vehicle once daily by oral gavage for three months. Accordingly, the present study should be regarded as a proof-of-concept investigation, whereas dose–response studies will be required to identify the optimal therapeutic dose. Diosmin was suspended in a 3% aqueous methylcellulose solution and administered by oral gavage in a final volume of 0.2 mL. Control animals received an equivalent volume of vehicle alone. After the treatment period, the animals were anaesthetized and sacrificed to collect tissues.

The investigational product used in this study was μsmin^®^ Plus (Giellepi S.p.A., Milan, Italy), a commercially available standardized diosmin-based ingredient (>80% diosmin; ≥90% total flavonoids; batch no. GLP01-2504001). The product was provided as a ready-to-use commercial formulation, and no extraction, isolation or purification of diosmin from plant material was performed by the authors.

### 2.2. Preparation of Small Mesenteric Arteries for Functional Experiments

After dissection, the first branch of mesenteric artery was placed in cold (4 °C) physiological salt solution (PSS) containing (in mmol/L): NaCl 120, NaHCO_3_ 25, KCl 4.7, KH_2_PO_4_ 1.18, MgSO_4_ 1.18, CaCl_2_ 2.5, EDTA 0.026 and glucose 5.5, as previously published [10]. A second-order branch of the mesenteric arterial tree (≈2 mm in length) was dissected and mounted on 2-glass microcannulas in a pressurized myograph, as previously described [10]. Vessels were equilibrated for 60 min under constant intraluminal pressure (45 mmHg) in warmed (37 °C) and bubbled (95% air and 5% CO_2_) PSS, at pH 7.4. Vessels were considered viable and used if they constricted >70% of their resting lumen diameter in response to an extraluminal application of high-potassium solution (125 mmol/L of KCl) containing 100 μmol/L of noradrenaline (NA).

An assessment of the vascular function was performed, as previously described [11]. Endothelium-dependent and -independent relaxations were assessed by cumulative concentrations of acetylcholine and sodium nitroprusside in vessels precontracted with norepinephrine. NO availability was investigated by repeating the dose–response curves to acetylcholine after incubation with the NO synthase inhibitor. Acetylcholine-induced vasorelaxation has been reported as the percentage change in vessel diameter relative to the preconstricted condition induced by noradrenaline. Media thickness and lumen diameter were measured at 3 different points from each small artery to obtain the media–lumen ratio (M/L). The media cross-sectional area (MCSA) was obtained by subtracting the internal from the external cross-sectional areas using outer plus lumen diameters, as previously described.

### 2.3. Assessment of Vascular DHE Fluorescence

The in situ production of superoxide anion from 30 mm frozen mesenteric vessel sections was evaluated at the confocal microscope by means of the fluorescent dye dihydroethidium [(DHE), Sigma], as previously described [12]. Three slides per segment were analyzed simultaneously after incubation with Krebs solution at 37 °C for 30 min. Krebs-HEPES buffer containing 2 μM DHE was then applied to each section and evaluated under fluorescence microscopy. In the presence of superoxide, DHE undergoes oxidation and intercalates in cell DNA, thus staining the nucleus with red fluorescence (excitation at 488 nm, emission 610 nm). Fluorescence is reported as the mean fluorescence intensity (MFI) of the arterial wall area stained with a red signal, which was evaluated using imaging software (McBiophotonics ImageJ, 1.54). MFI values for each sample were normalized to the mean MFI of the control group and expressed as a percentage of the controls. Since selective enzymatic inhibitors and direct measurements of NADPH oxidase activity were not employed, DHE staining was not used to identify the specific enzymatic source of the detected oxidative signal.

### 2.4. MitoSOX Red Staining

The level of mitochondrial ROS was determined by staining mesenteric arteries with MitoSOX Red dye (Life Technologies, Thermo fisher Scientific, Waltham, MA USA). Briefly, mesenteric arteries were washed 3 times with PBS buffer and then loaded with MitoSOX Red in the dark at a temperature of 37 °C for 30 min. A fluorescence microscope (Carl Zeiss, Jena, Germany) was used to detect fluorescent signals with an excitation wavelength at 510 nm and emission wavelength at 580 nm. Fluorescence is reported as the MFI of the arterial wall area stained with a red signal, which was evaluated using imaging software (McBiophotonics ImageJ, 1.54). MFI values for each sample were normalized to the mean MFI of the control group and expressed as a percentage of the controls.

### 2.5. Plasma Levels of TNF and MDA

Serum levels of TNF and MDA were analyzed by ELISA kit (Prodotti Gianni, Milan, Italy), as previously described [13]. Aliquots of 100 μL were used for the test.

For the evaluation of MDA plasma levels, a colorimetric assay kit (Nordic Biosite, Stockholm, Sweden) was employed and performed following the manufacturer’s instructions. MDA and TNF levels were indicated as nanograms per milliliter (ng/mL) of serum.

### 2.6. Evaluation of Lipopolysaccharide-Binding Protein (LBP) Levels in Plasma

Blood samples were centrifuged for 5 min at 4000 *g* at 2–8 °C, and after the centrifugation, supernatants were collected. Aliquots (100 μL) were used for the assay. LBP levels were expressed as picograms per milliliter (ρg/mL) of plasma.

### 2.7. Real-Time qPCR

Total RNA was extracted using TRIzol Reagent (Invitrogen, Carlsbad, CA, USA) according to the manufacturer’s recommendations. Before extraction, vascular samples were manually lysed using Microtube Pestles (Starlab, Milan, Italy) and then sonicated via Ultrasonic Processor (Merck, Milan, Italy).

Conversion of total cellular RNA to cDNA was carried out using the High-Capacity cDNA Reverse Transcription Kit (Thermo Fisher Scientific, Milan, Italy), according to the manufacturer’s instructions. Real-time quantitative PCR was performed to assess the expression of nitric oxide synthase 3 (*Nos3*), superoxide dismutase 2 (*Sod2*), xanthine dehydrogenase (*Xdh*) and glutathione peroxidase (*Gpx1*). *Gapdh* was used as the endogenous reference gene (Table 1). Real-time PCR reactions were performed using SYBR Select Master Mix (Applied Biosystems, Thermo Fisher Scientific, Milan, Italy) on a CFX Duet Real-Time PCR System (Bio-Rad, Milan, Italy), according to the manufacturer’s instructions. The amplification program consisted of 1 cycle at 95 °C for 10 min, followed by 40 cycles with a denaturing phase at 95 °C for 30 s and an annealing and elongation phase of 1 min at 60 °C. A melting curve analysis was performed after amplification to verify the accuracy of the amplicon. Differences in Ct values between test genes and endogenous controls (ΔCt) and between different groups and control group (ΔΔCt) used as negative power of 2 (2^−ΔΔCt^) were calculated and used for statistical analysis.

### 2.8. Statistical Analysis

The experimental unit was the individual animal. Data are presented as the mean ± SEM or as violin plots showing individual values and were analyzed using GraphPad Prism 7.0 (GraphPad Software LLC, Boston, MA, USA). For outcomes measured across the four experimental groups, data were analyzed by two-way ANOVA, with age and diosmin treatment as fixed factors. When required by the structure of the dataset, a linear mixed-effects model fitted by restricted maximum likelihood (REML), including age, treatment and their interaction, was applied. Fisher’s least significant difference (LSD) post hoc test was used for the prespecified pairwise comparisons. Comparisons involving only two independent groups were performed using an unpaired two-tailed Student’s *t*-test. A *p* value < 0.05 was considered statistically significant.

## 3. Results

### 3.1. Isolation of Mesenteric Arteries and In Vitro Assessment of Their Structure and Reactivity

**Figure 1:**
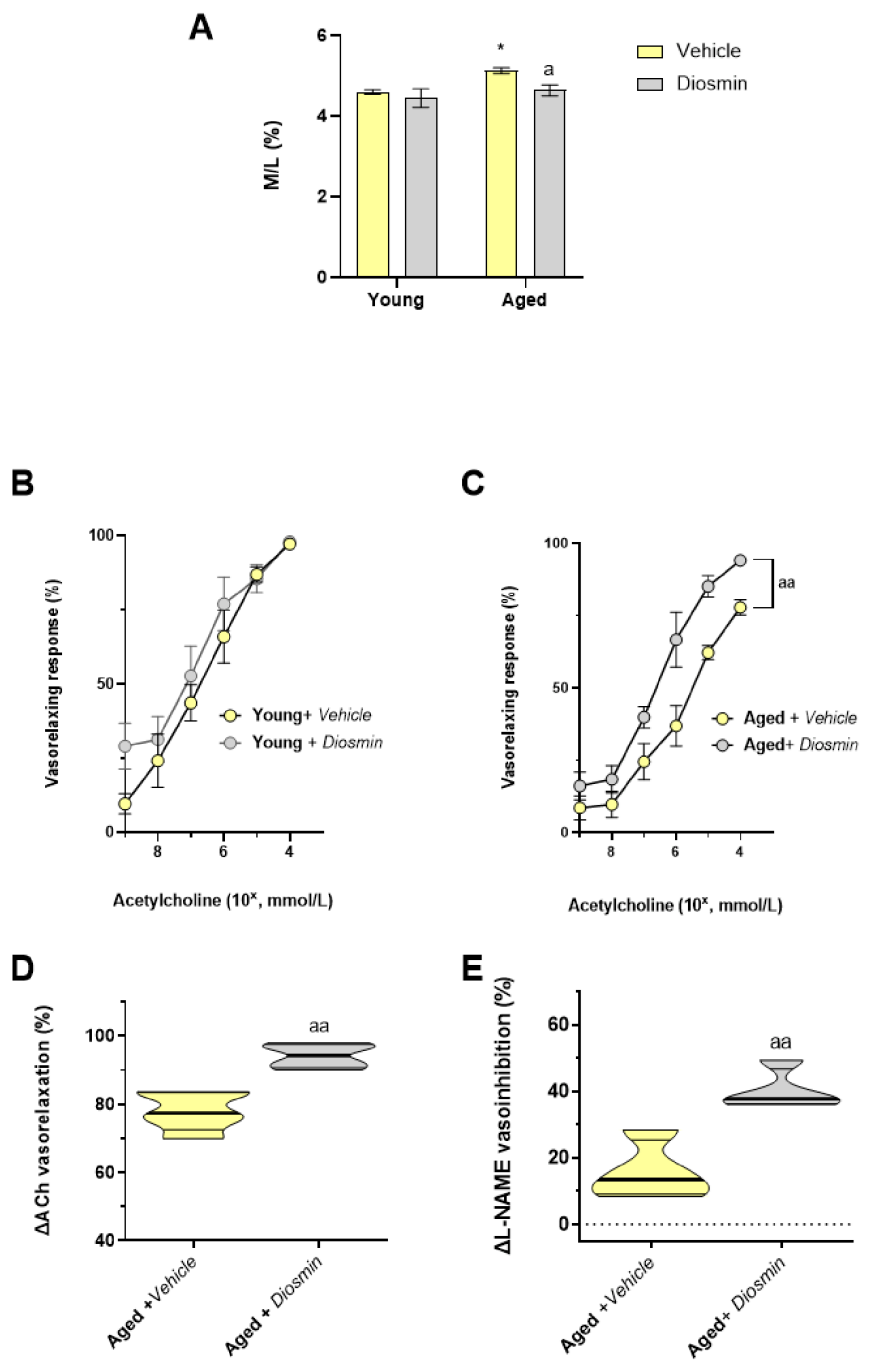
Effects of chronic diosmin supplementation on microvascular structure and endothelial function. (**A**) Media-to-lumen ratio (M/L), expressed as a dimensionless ratio, as an index of vascular remodeling. Statistical analysis was performed using a linear mixed-effects model (REML), followed by Fisher’s least significant difference (LSD) post hoc test. (**B**,**C**) Concentration–response curves to acetylcholine in young (**B**) and aged (**C**) rats. Vasorelaxation is expressed as the percentage change in vessel diameter relative to the noradrenaline-precontracted condition. (**D**) Maximal acetylcholine-induced vasorelaxation in aged rats. (**E**) L-NAME-sensitive component of acetylcholine-induced vasorelaxation, expressed as the percentage inhibition of maximal acetylcholine relaxation, as an index of NO-dependent endothelial function. Data from panels D and E were analyzed using an unpaired two-tailed Student’s *t*-test. Data are presented as mean ± SEM (**A**–**C**) or as violin plots (**D**,**E**). * *p* < 0.05 versus the young vehicle-treated group; a, *p* < 0.05 and aa, *p* < 0.01 versus the aged vehicle-treated group. *n* = 4–5 animals per group.

No differences in vascular remodeling were observed in young rats, although an improvement in media/lumen ratio was observed in aged rats treated with diosmin. Moreover, we observed an increase in aged rats administered with vehicle, compared to non-treated young rats (Figure 1A). In vascular relaxation assays, no differences between treatment groups were observed in young rats (Figure 1B). In aged rats, chronic supplementation with diosmin alone induced a partial restoration of vascular relaxation to ACh (Figure 1C,D), also in terms of L-NAME vasoinhibition (Figure 1E), a reliable marker of endothelial function. Taken together, our data indicate that chronic diosmin supplementation is able to partially rescue the vascular phenotype in aged rats.

### 3.2. Vascular DHE Fluorescence and Mitochondrial Reactive Oxygen Species

**Figure 2:**
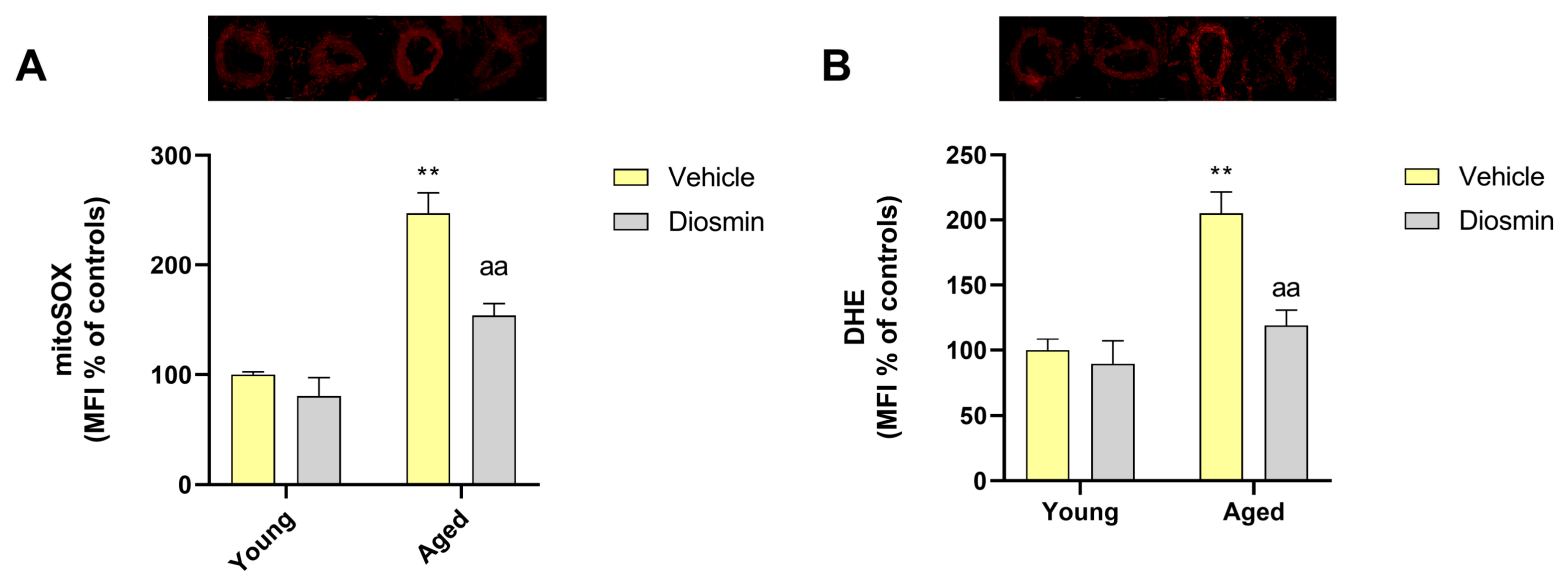
Effects of chronic diosmin supplementation on vascular oxidative stress. Representative fluorescence images and quantitative analysis of mitochondrial superoxide (MitoSOX fluorescence, (**A**) and vascular DHE fluorescence (**B**) in mesenteric resistance arteries. Representative images were acquired using a 20–40× objective, and the scale bar represents 20 μm. Fluorescence intensity was quantified as mean fluorescence intensity (MFI) using ImageJ software, normalized to the mean value of the young vehicle-treated group and expressed as percentage of control. Statistical analysis was performed by two-way ANOVA (age × treatment), followed by Fisher’s least significant difference (LSD) post hoc test. Data are presented as mean ± SEM (*n* = 3 animals per group). **, *p* < 0.01 versus the young vehicle-treated group; aa, *p* < 0.01 versus the aged vehicle-treated group.

The fluorescence assays showed that in the vascular wall of young rats no differences were detected in terms of mtROS (assessed via mitoSOX staining) or vascular oxidative signal detected by DHE between untreated and diosmin-treated animals. In aged rats administered with vehicle, the levels of mtROS and DHE were significantly higher, as compared with young non-treated animals. In this setting, chronic supplementation with diosmin was able to mitigate the mtROS and vascular oxidative signal detected by DHE. In fact, aged animals administered with diosmin showed a significant decrease in mtROS and DHE expressions, compared with non-treated aged animals (Figure 2A,B). This further confirmed our functional data, showing that chronic diosmin supplementation is able to partially mitigate the reactive oxygen species levels in the vascular wall of aged rats.

### 3.3. Evaluation of Plasma Inflammatory Parameter (TNF) and Oxidative Stress Index (MDA)

**Figure 3:**
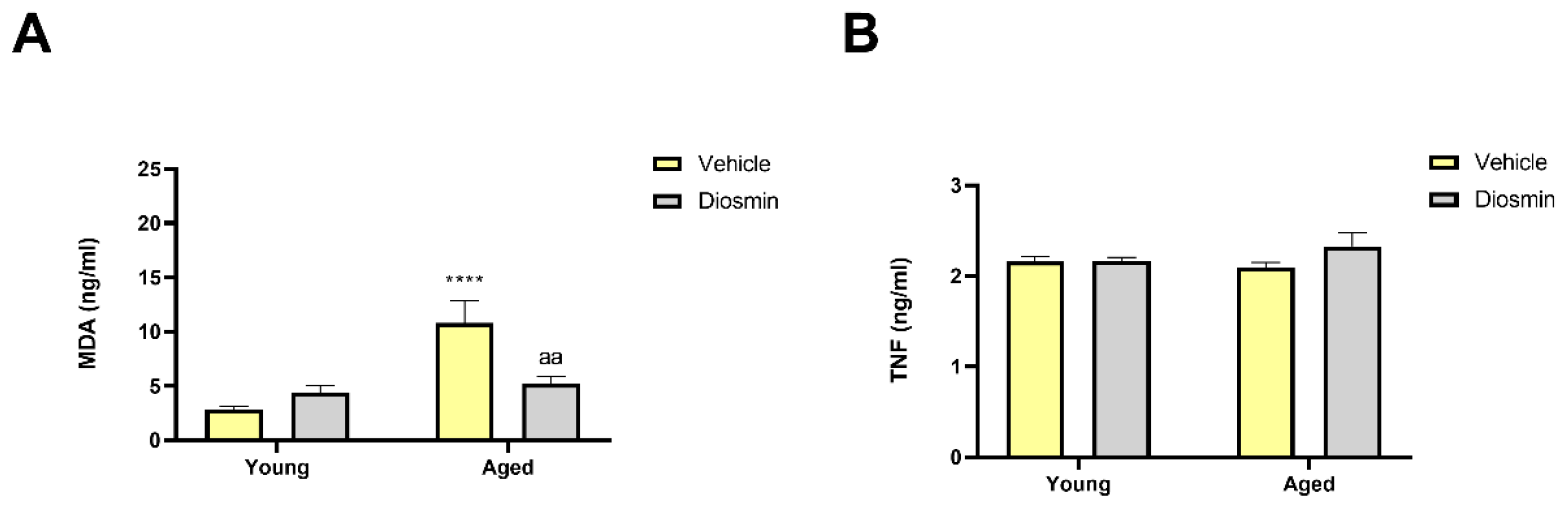
Effects of chronic diosmin supplementation on systemic oxidative stress and circulating TNF levels. (**A**) Plasma malondialdehyde (MDA) concentrations (ng/mL). (**B**) Plasma TNF concentrations (ng/mL). Statistical analysis was performed by using a linear mixed-effects model (REML), followed by Fisher’s least significant difference (LSD) post hoc test. Data are presented as mean ± SEM (*n* = 6–8 animals per group). **** *p* < 0.0001 versus the young vehicle-treated group; aa, *p* < 0.01 versus the aged vehicle-treated group.

The MDA levels in plasma from aged animals administered with vehicle were significantly increased, as compared with young animals administered with vehicle (Figure 3A). Administration of aged animals with diosmin significantly decreased the MDA, compared to aged animal treated with vehicle. No significant changes were observed in the TNF levels (Figure 3B) in young and aged rats treated with vehicle or diosmin.

### 3.4. Evaluation of Plasma LBP Levels

**Figure 4:**
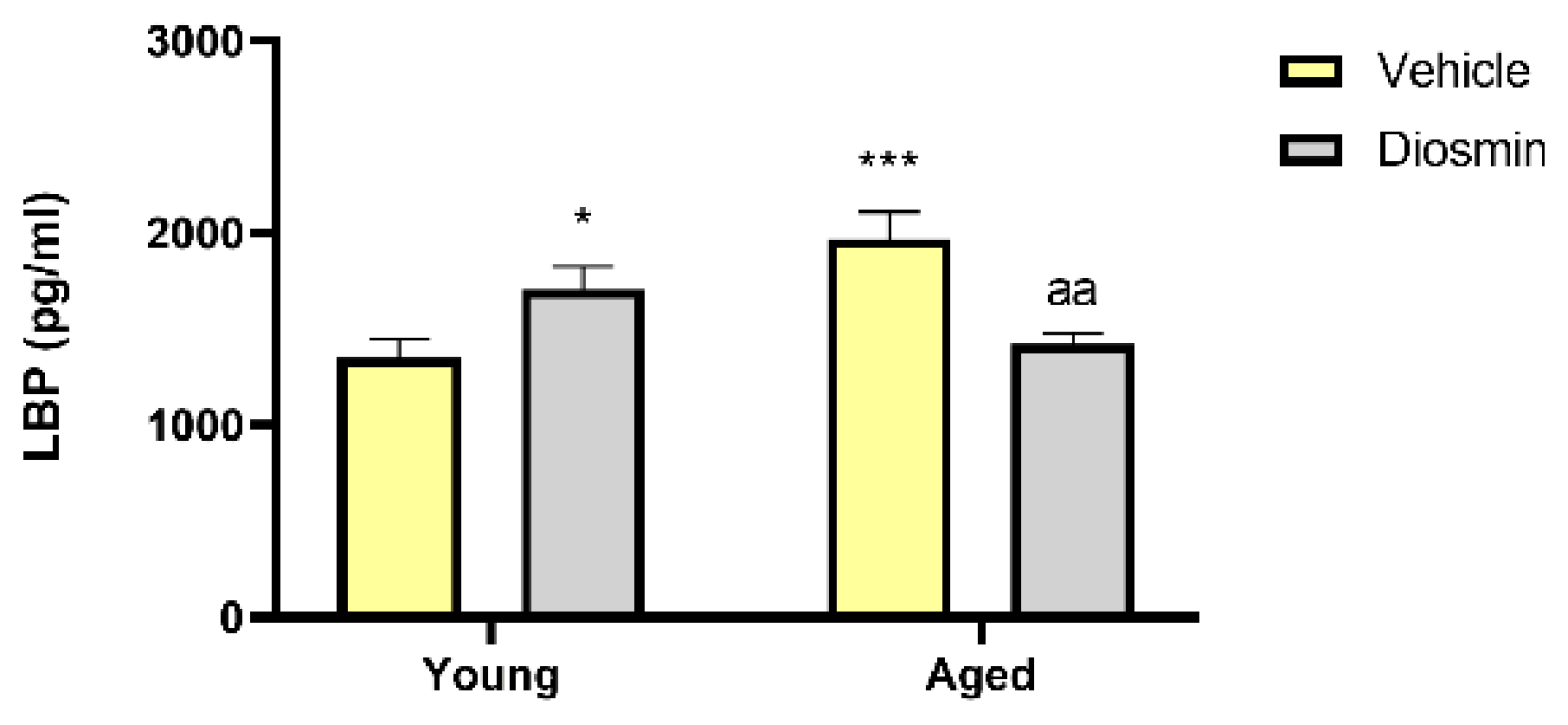
Effects of chronic diosmin supplementation on circulating lipopolysaccharide-binding protein (LBP). Plasma LBP concentrations (pg/mL) in young and aged rats treated with vehicle or diosmin for 3 months. Statistical analysis was performed by linear mixed-effects model (REML), followed by Fisher’s least significant difference (LSD) post hoc test. Data are presented as mean ± SEM (*n* = 5–8 animals per group). * *p* < 0.01 and ***, *p* < 0.001 versus the young vehicle-treated group; aa, *p* < 0.01 versus the aged vehicle-treated group.

The LBP levels in plasma from aged animals administered with vehicle were significantly enhanced compared to young animals administered with vehicle (Figure 4). Administration of aged animals with diosmin significantly reduced this parameter. Moreover, the LBP plasma levels were significantly enhanced in young animals administered with diosmin, compared to young treated with vehicle.

### 3.5. Nos3, Sod2, Gpx and Xdh Gene Expression in Vascular Tissues

**Figure 5:**
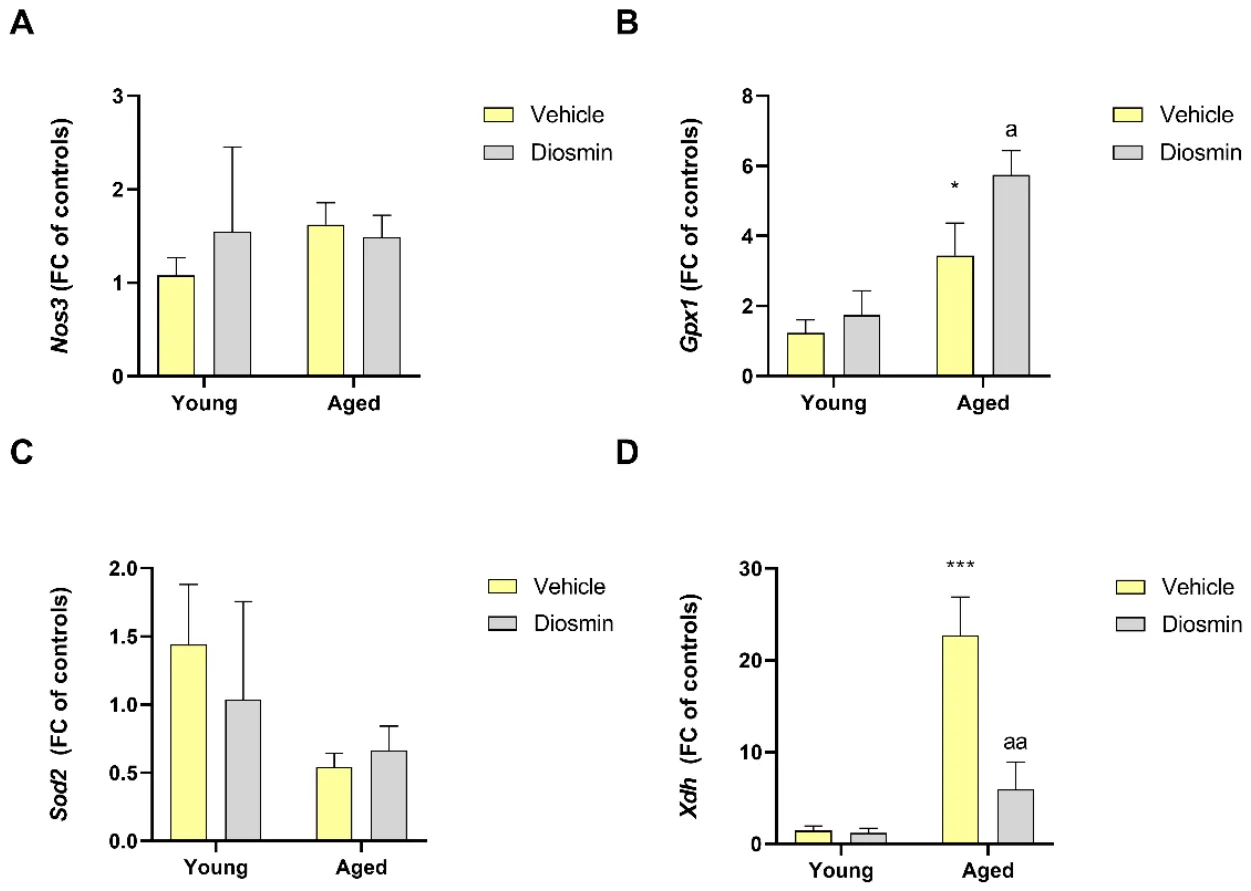
Effects of chronic diosmin supplementation on vascular gene expression. Relative mRNA expression of *Nos3* (**A**), *Gpx1* (**B**), *Sod2* (**C**) and *Xdh* (**D**) in mesenteric resistance arteries. Gene expression was determined by quantitative real-time PCR using the 2^−ΔΔCt^ method, normalized to Gapdh and expressed relative to the young vehicle-treated group. Statistical analysis was performed by two-way ANOVA (age × treatment) or linear mixed-effects model (REML), followed by Fisher’s least significant difference (LSD) post hoc test. Data are presented as mean ± SEM (*n* =4–7 animals per group). *, *p* < 0.05 and ***, *p* < 0.001 versus the young vehicle-treated group; a, *p* < 0.05 and aa, *p* < 0.01 versus the aged vehicle-treated group.

No significant differences were observed in *Nos3* expression among groups, with diosmin treatment in aged animals showing no detectable effect compared to the untreated aged controls (Figure 5A). The *Gpx1* expression significantly increased in aged animals treated with diosmin compared to untreated aged rats; moreover, aged rats administered with vehicle showed a significant increase in *Gpx1* expression, compared with young administered with vehicle (Figure 5B). Similarly, *Sod2* levels were not significantly altered by diosmin treatment in either the young or aged groups (Figure 5C), indicating no major effect on this antioxidant enzyme.

In contrast, the *Xdh* expression was markedly elevated in aged animals compared to young controls, and this increase was significantly reduced by diosmin treatment in the aged group (Figure 5D), suggesting a potential modulatory effect of diosmin on age-associated oxidative stress pathways.

## 4. Discussion

The present study provides novel evidence that chronic diosmin supplementation exerts protective vascular effects in a rat model of vascular inflammaging. Specifically, diosmin partially restored endothelial function and improved vascular wall structure in aged animals, while attenuating oxidative stress-related alterations and inflammatory markers. These findings support the concept that diosmin effectively targets key pathophysiological mechanisms underlying vascular aging, including endothelial dysfunction, reactive oxygen species (ROS) accumulation and chronic low-grade inflammation [1].

Vascular aging is a complex multifactorial process characterized by structural remodeling, endothelial impairment and increased arterial stiffness, all of which contribute to the heightened cardiovascular risk observed in elderly populations. A growing body of evidence identifies inflammaging, a persistent low-grade inflammatory state, as a central driver of these alterations, promoting oxidative stress, impairing nitric oxide (NO) bioavailability and fostering maladaptive vascular remodeling [1,14]. In this context, our findings suggest that diosmin interrupts, at least in part, the self-reinforcing interplay between the oxidative stress and inflammation that sustains vascular aging.

Endothelial dysfunction represents one of the earliest and most clinically relevant manifestations of vascular aging. In the present study, aged animals exhibited impaired acetylcholine (ACh)-induced vasorelaxation. Chronic diosmin supplementation significantly improved endothelium-dependent relaxation, as evidenced by the partial restoration of ACh responses and modulation of the L-NAME-sensitive component of the vasorelaxation response. The higher contribution of the NOS-dependent component to acetylcholine-induced relaxation in diosmin-treated aged vessels is consistent with a possible preservation of NO-mediated endothelial function [11,15]. However, because NO concentration, NO metabolites and eNOS activity were not assessed, increased NO bioavailability cannot be demonstrated directly. Therefore, the present findings should be interpreted as functional evidence of improved endothelial responsiveness rather than direct evidence of enhanced NO production or bioavailability.

Mechanistically, previous studies have demonstrated that diosmin inhibits nuclear factor-κB (NF-κB) signaling and reduces the expression of pro-inflammatory cytokines such as TNF, IL-1β and IL-6 [7,8]. Given that NF-κB activation negatively regulates *Nos3* function and promotes endothelial activation, suppression of this pathway likely contributes to the improved vascular responses observed in the present study. In parallel, the well-documented antioxidant properties of diosmin, including free radical scavenging and the inhibition of lipid peroxidation, may further enhance NO bioavailability by limiting the formation of peroxynitrite and other reactive nitrogen species [7,8].

A key finding of this study is the reduction in vascular DHE fluorescence and mitochondrial MitoSOX fluorescence following diosmin treatment in aged animals. These findings indicate an attenuation of vascular oxidative stress and are consistent with the improvement in endothelial function observed in the functional experiments. Oxidative stress arises from multiple cellular sources, including mitochondrial dysfunction and several ROS-generating enzymatic systems, such as NADPH oxidases [16,17]. The reduction in DHE and MitoSOX fluorescence suggests that diosmin attenuates the overall vascular oxidative burden through an integrated antioxidant effect. However, DHE fluorescence does not allow identification of the specific enzymatic source responsible for the detected oxidative signal. Since selective NADPH oxidase inhibitors, NOX isoform expression or direct measurements of NADPH oxidase activity were not included in the present study, the reduction in DHE fluorescence should not be interpreted as direct evidence of NADPH oxidase inhibition. Rather, it indicates an overall attenuation of vascular oxidative stress.

The molecular pathways underlying these effects remain incompletely defined; however, flavonoids such as diosmin and its aglycone diosmetin are known to modulate redox-sensitive transcription factors, including NF-κB and Nrf2, thereby influencing the expression of endogenous antioxidant systems [8]. Consistent with this framework, diosmin did not significantly modify the expression of canonical antioxidant enzymes, suggesting that its antioxidant effects are unlikely to depend on transcriptional upregulation of these pathways. By simultaneously reducing ROS production and enhancing antioxidant capacity, diosmin likely disrupts the feed-forward loop linking oxidative stress, mitochondrial dysfunction and vascular inflammation [18]. Beyond vascular inflammaging, diosmin has been implicated in modulation of endothelial homeostasis, mitochondrial function, lipid peroxidation, redox-sensitive transcriptional networks and gut barrier-associated signaling. Although these pathways were not directly investigated in the present study, they may contribute to the broader biological effects of diosmin and deserve further investigation.

In line with these observations, the transcriptional profiling of oxidative stress-related genes revealed a selective pattern of modulation by diosmin. While the expression of *Nos3*, *Sod2* and *Gpx1* remained largely unchanged across experimental groups, aging was associated with a marked upregulation of *Xdh*, which was significantly attenuated by diosmin treatment. Importantly, the lack of significant changes in *Nos3*, *Sod2* and *Gpx1* mRNA expression does not exclude the possible effects of diosmin on protein abundance, post-translational modifications, subcellular localization or enzymatic activity. Since these parameters were not evaluated in the present study, the current findings should be interpreted as evidence of unchanged transcript levels only. Given the well-established role of *Xdh/xanthine oxidase* as a major enzymatic source of superoxide and hydrogen peroxide in the vasculature [19], its downregulation suggests that diosmin preferentially targets upstream ROS-generating systems rather than broadly inducing antioxidant gene expression. This interpretation is further supported by the absence of compensatory upregulation of canonical antioxidant enzymes, indicating that the observed redox improvement is unlikely to be driven by enhanced scavenging capacity. Instead, diosmin appears to mitigate oxidative stress at its source, thereby reducing the overall oxidative burden [20]. Importantly, the lack of transcriptional changes in *Nos3*, despite clear functional improvement in endothelium-dependent vasorelaxation, strongly supports the hypothesis that diosmin may preserve NO-mediated endothelial function primarily by limiting ROS-mediated NO degradation, rather than by increasing *Nos3* expression per se. Collectively, these findings provide mechanistic evidence that diosmin exerts vascular protection in aging through targeted suppression of pro-oxidant pathways, effectively uncoupling the feed-forward loop between ROS overproduction and endothelial dysfunction that characterizes vascular inflammaging.

Consistent with the systemic presence of inflammaging, aged animals displayed elevated plasma levels of malondialdehyde (MDA), a marker of lipid peroxidation, as well as increased lipopolysaccharide-binding protein (LBP), indicative of metabolic endotoxemia and impaired gut barrier function [21,22]. Diosmin treatment significantly reduced the MDA and LBP levels, supporting its combined antioxidant and anti-inflammatory effects at the systemic level.

An unexpected finding was the modest increase in circulating LBP observed in young diosmin-treated animals compared with young vehicle-treated controls. Because this change occurred in healthy animals lacking evidence of vascular dysfunction or systemic oxidative stress, it is unlikely to reflect impairment of intestinal barrier integrity. Instead, it may represent an adaptive physiological response associated with polyphenol administration. Plant polyphenols have been proposed to induce hormetic responses, characterized by transient activation of cellular stress-response pathways that ultimately enhance tissue resilience rather than promote pathology [6]. In addition, inter-individual variability in gut microbiota composition may influence the metabolism and biological effects of flavonoids, contributing to heterogeneous responses in healthy animals [6,23]. Although this interpretation remains speculative, it is consistent with the opposite effect observed in aged animals, where diosmin significantly reduced circulating LBP, suggesting that its effects on gut barrier-associated inflammatory signaling may depend on the underlying biological context. In contrast, the reduction in circulating LBP observed in aged animals integrates with the suppression of Xdh expression and vascular ROS production, providing a coherent multi-level mechanism underlying diosmin protective effects. LBP is a surrogate marker of endotoxin exposure and gut-derived inflammation [22], which represent important upstream triggers of systemic oxidative stress and endothelial dysfunction. Thus, the decrease in LBP suggests that diosmin may attenuate circulating pro-inflammatory stimuli that contribute to the activation of vascular ROS-generating systems, including *Xdh*/*xanthine oxidase* pathways. In this framework, reduced metabolic endotoxemia may alleviate redox-sensitive signaling cascades, ultimately limiting ROS overproduction and preserving nitric oxide bioavailability [24]. These findings support an integrated model in which diosmin acts both locally, by suppressing vascular pro-oxidant enzymes, and systemically, by reducing gut-derived inflammatory inputs, thereby disrupting the bidirectional amplification between oxidative stress and inflammation that characterizes vascular inflammaging. Although direct assessment of intestinal permeability or microbiota composition was not performed, this interpretation is consistent with previous evidence indicating that flavonoids can modulate gut barrier function and host–microbiota interactions [25,26].

Although diosmin significantly reduced circulating LBP levels, this finding should not be interpreted as conclusive evidence of the suppression of chronic inflammation. Rather, it indicates a reduction in one circulating marker associated with endotoxin exposure. The unchanged circulating TNF concentration, together with the absence of additional circulating or vascular inflammatory markers, limits conclusions regarding the anti-inflammatory effects of diosmin in the present model. Therefore, while the reduction in LBP is consistent with a possible modulation of systemic inflammatory inputs, broader inflammatory profiling will be required to establish whether diosmin directly modulates vascular inflammaging. This observation also suggests that circulating TNF may not adequately reflect the complexity of inflammaging in this model and that tissue-specific inflammatory pathways may be more sensitive to diosmin treatment than systemic cytokine concentrations. It is plausible that diosmin exerts its primary anti-inflammatory effects at the tissue level, where the modulation of redox-sensitive pathways and endothelial activation occurs independently of overt systemic cytokine suppression.

The reduction in LBP is particularly noteworthy, as increased circulating LBP in aging has been associated with translocation of bacterial endotoxins from the gut, contributing to systemic inflammation and vascular dysfunction. Diosmin has been reported to improve intestinal barrier integrity and modulate gut microbiota composition [27], suggesting that its vascular protective effects may, in part, be mediated through modulation of the gut–vascular axis. This observation highlights a potential multi-organ mechanism of action, integrating metabolic, inflammatory and vascular pathways.

Structural remodeling of the vasculature is another hallmark of aging. In the present study, aged animals exhibited an increased media-to-lumen ratio in mesenteric resistance arteries, indicative of hypertrophic remodeling. Chronic diosmin supplementation partially reversed these structural alterations, suggesting a protective effect on vascular architecture. These improvements may result from reduced oxidative injury to vascular smooth muscle cells and attenuation of pro-fibrotic signaling pathways, including those mediated by TGF-β and matrix metalloproteinases, both of which are regulated by redox-sensitive mechanisms [28].

Taken together, our findings support a mechanistic model in which diosmin disrupts the reciprocal amplification between the oxidative stress and inflammation that drives vascular aging. Excess ROS production from mitochondrial dysfunction and NADPH oxidase activation promotes inflammatory signaling, endothelial dysfunction, and structural remodeling, establishing a self-perpetuating cycle of vascular damage [29]. Importantly, diosmin’s mechanism of action likely involves both direct radical scavenging and transcriptional regulation of redox-sensitive genes. Activation of the Nrf2 pathway represents a plausible unifying mechanism, potentially accounting for both enhanced antioxidant defenses and improved vascular phenotype [30], and warrants further investigation.

Diosmin is already widely used in clinical practice for chronic venous insufficiency and hemorrhoidal disease due to its venotonic and anti-inflammatory properties [8]. The present findings expand its therapeutic relevance, suggesting that diosmin may be repurposed for the prevention or treatment of vascular aging and related cardiovascular disorders. Its ability to simultaneously target oxidative stress, inflammation, and endothelial dysfunction positions it as a promising candidate among nutraceutical and pharmacological strategies aimed at modulating inflammaging.

Similar vascular protective effects have been reported for other flavonoids, including quercetin and hesperidin; however, diosmin may offer pharmacokinetic advantages, such as improved oral bioavailability and a longer plasma half-life, potentially enhancing its efficacy in chronic settings [31,32]. In this regard, previous pharmacokinetic studies have shown that the diosmin-based formulation investigated in this study exhibits superior bioavailability compared with micronized diosmin in both rats and humans [33]. These considerations, together with the mechanistic insights provided here, support further translational investigation. Nevertheless, direct head-to-head comparisons between diosmin and other flavonoids or non-flavonoid endothelial-protective agents have not yet been performed under comparable experimental conditions. Such studies will be necessary to establish whether the vascular effects observed here are unique to diosmin or represent a broader class effect of antioxidant compounds.

However, several limitations should be acknowledged. First, only a single dose of diosmin was evaluated. Moreover, the endothelial function was evaluated only at the end of the three-month treatment period. Consequently, the temporal onset of diosmin-induced vascular protection cannot be established and should be investigated in future time-course studies. The study was designed to investigate the long-term vascular effects of a previously established biologically active dose rather than to perform a dose-ranging assessment. Therefore, the minimum effective dose, the dose–response relationship and the potential existence of a ceiling effect cannot be established. In addition, plasma and tissue concentrations of diosmin and its active metabolite diosmetin were not measured, precluding direct pharmacokinetic–pharmacodynamic correlations. Second, the present study was conducted exclusively in male animals, and sex-dependent differences in vascular aging and flavonoid responsiveness cannot be excluded. Third, protein expression, post-translational modifications and the enzymatic activity of eNOS, SOD2, GPx1 and XDH were not assessed. Therefore, the absence of transcriptional changes cannot be interpreted as evidence of unchanged protein abundance or biological activity. Furthermore, although functional and biochemical improvements were clearly demonstrated, the specific molecular mediators of diosmin action, including NF-κB inhibition, Nrf2 activation and modulation of individual NOX isoforms, were not directly evaluated. Future studies incorporating protein expression and mechanistic analyses will be essential to clarify these pathways. Finally, the inflammatory characterization was restricted to circulating TNF and LBP. Vascular cytokine expression, immune-cell infiltration, endotoxin concentrations and a broader systemic inflammatory profile were not assessed. Consequently, the present findings primarily support an antioxidant mechanism associated with improved vascular function, whereas conclusions regarding direct modulation of vascular inflammaging remain exploratory. Although DHE and MitoSOX provide valuable in situ information on vascular oxidative stress, complementary approaches such as electron paramagnetic resonance spectroscopy, HPLC-based characterization of DHE oxidation products, direct quantification of hydrogen peroxide or assessment of NADPH oxidase and xanthine oxidase activity could further strengthen the mechanistic interpretation.

## 5. Conclusions

This study demonstrates that chronic diosmin supplementation improves the vascular structure and function in aged animals while attenuating vascular oxidative stress and selected features associated with vascular inflammaging. These effects were associated with reduced ROS generation from both vascular and mitochondrial sources, restoration of endothelial function and a reduction in systemic oxidative and inflammatory markers. Collectively, these findings support a model in which diosmin interrupts the pathogenic cycle linking oxidative stress, inflammation and vascular dysfunction, thereby slowing the progression of vascular aging. Given its established safety profile and clinical use, diosmin emerges as a promising candidate for targeting age-related vascular disorders. However, the extent to which these effects may be shared by other diosmin formulations remains to be established. Further studies are warranted to validate these findings in human populations and to elucidate the molecular pathways underlying its protective effects.

## Figures and Tables

**Table 1:** Primer sequences and expected amplicon sizes used for RT-qPCR analysis.

*Gene Symbol*	*Primer*	*Accession Number*	*Amplicon Length*
*Nos3*	Fw: GGGCCAGGGTGATGAGCTCTG	NM_021838.2	324
	Rv: CCCTCCTGGCTTCCAGTGTCC		
*Sod2*	Fw: CGCTGGAGCCGCACATTAAC	NM_017051.2	70
	Rv: TTCACGTAGGTCGCGTGGTG		
*Xdh*	Fw: AGCCACGATGACTGCGGATG	NM_017154.2	128
	Rv: GCTTGGTCCCACATAGCCCC		
*Gpx1*	Fw: AGTTCGGACATCAGGAGAATGGCA	NM_030826.4	121
	Rv: TCACCATTCACCTCGCACTTCTCA		
*Gapdh*	Fw: CATCACTGCCACCCAGAAGACTG	NM_017008.4	153
	Rv: ATGCCAGTGAGCTTCCCGTTCAG		

## Data Availability

The raw data supporting the conclusions of this article will be made available by the authors on request.

## Abbreviations

The following abbreviations are used in this manuscript:

Ach	Acetylcholine
ANOVA	Analysis Of Variance
cDNA	Complementary DNA
Ct	Cycle Threshold
DHE	Dihydroethidium
EDTA	Ethylenediaminetetraacetic Acid
ELISA	Enzyme-Linked Immunosorbent Assay
*Nos3*	Endothelial Nitric Oxide Synthase
*Gapdh*	Glyceraldehyde-3-Phosphate Dehydrogenase
*GPx*	Glutathione Peroxidase
IFN	Interferon
IL	Interleukin
LBP	Lipopolysaccharide-Binding Protein
L-NAME	N(Ω)-Nitro-L-Arginine Methyl Ester
MDA	Malondialdehyde
mtROS	Mitochondrial Reactive Oxygen Species
NA	Noradrenaline
NADPH	Nicotinamide Adenine Dinucleotide Phosphate
NF-κB	Nuclear Factor Kappa B
NO	Nitric Oxide
NOX	NADPH Oxidase
Nrf2	Nuclear Factor Erythroid 2-Related Factor 2
PBS	Phosphate-Buffered Saline
PCR	Polymerase Chain Reaction
PSS	Physiological Salt Solution
RNA	Ribonucleic Acid
ROS	Reactive Oxygen Species
SEM	Standard Error of The Mean
*Sod2*	Superoxide Dismutase 2
TGF-β	Transforming Growth Factor Beta
TNF	Tumor Necrosis Factor
*Xdh*	Xanthine Dehydrogenase
